# IMGMD: A platform for the integration and standardisation of *In silico* Microbial Genome-scale Metabolic Models

**DOI:** 10.1038/s41598-017-00820-6

**Published:** 2017-04-07

**Authors:** Chao Ye, Nan Xu, Chuan Dong, Yuannong Ye, Xuan Zou, Xiulai Chen, Fengbiao Guo, Liming Liu

**Affiliations:** 1 State Key Laboratory of Food Science and Technology, Jiangnan University, 1800 Lihu Road, Wuxi, Jiangsu 214122 China; 2 Key Laboratory for Neuroinformation of Ministry of Education, University of Electronic Science and Technology of China, No. 4, 2nd Section, North Jianshe Road, Chengdu, Sichuan 610054 China; 3 The Key Laboratory of Industrial Biotechnology, Ministry of Education, Jiangnan University, 1800 Lihu Road, Wuxi, Jiangsu 214122 China; 4 School of Biology and Engineering, Guizhou Medical University, Dongqing Road, Huaxi District, Guiyang, Guizhou 550025 China; 5 School of Big Health, Guizhou Medical University, Dongqing Road, Huaxi District, Guiyang, Guizhou 550025 China

## Abstract

Genome-scale metabolic models (GSMMs) constitute a platform that combines genome sequences and detailed biochemical information to quantify microbial physiology at the system level. To improve the unity, integrity, correctness, and format of data in published GSMMs, a consensus IMGMD database was built in the LAMP (Linux + Apache + MySQL + PHP) system by integrating and standardizing 328 GSMMs constructed for 139 microorganisms. The IMGMD database can help microbial researchers download manually curated GSMMs, rapidly reconstruct standard GSMMs, design pathways, and identify metabolic targets for strategies on strain improvement. Moreover, the IMGMD database facilitates the integration of wet-lab and *in silico* data to gain an additional insight into microbial physiology. The IMGMD database is freely available, without any registration requirements, at http://imgmd.jiangnan.edu.cn/database.

## Introduction

Genome-scale metabolic models (GSMMs) are a kind of a mathematical model that integrates multiple types of omics data, such as genomics, transcriptomics, proteomics, and metabolomics. GSMMs can clarify the relations among genes, proteins, and reactions. Models can be used to describe all biochemical reactions, metabolites, and genes involved in the metabolism of a specific organism. They have been used to decipher metabolic, regulatory, and signaling networks at the whole-organism level^1–3^. Since the first GSMM of *Haemophilus influenzae* Rd was constructed in 1999^4^, more than 300 GSMMs for over 100 organisms have been built^5, 6^.

GSMMs have been developed for over 15 years, and four steps are involved in their construction: creation of a draft model, manual refinement, conversion to a mathematical format, and network evaluation^6–9^. Nonetheless, owing to differences in nomenclature^10^, integrity, and correctness^11^ as well as the format^12^ of published GSMMs, these models cannot be directly applied by other researchers. To reduce the manual labour needed for model construction and to increase the quality of GSMMs, some databases and tools, such as BIGG Models^11^ and MetaNetX^13^, have been developed. Nonetheless, they are limited by the quality and quantity of models. For example, BIGG Models includes only 80 high-quality GSMMs (as of 30 November 2016), which is far from the number of published models. In MetaNetX, only 24 (14.7%) models have been published, which were validated by experimental results (http://www.metanetx.org/cgi-bin/mnxweb/repository).

In this study, we built a database named *In silico* Microbial Genome-scale Metabolic Models (IMGMD) in the LAMP (Linux + Apache + MySQL + PHP) system. It provides a platform for integration and standardisation of all published microbial GSMMs. In IMGMD, users are not only able to browse and download standardised GSMMs but can also reconstruct GSMMs automatically. In addition to pathway mining and mutation library functions, users can access information that can guide pathway design and metabolic target identification.

## Results and Discussion

### Database content and web interface

**Figure 1:**
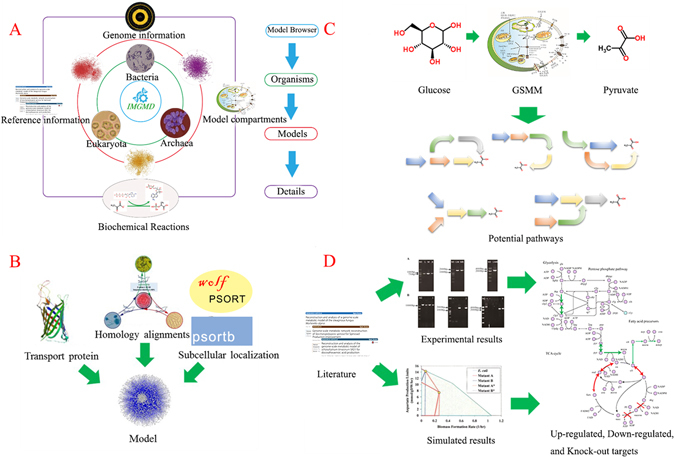
Summary of four functional modules in IMGMD. (**A**) The ‘model browser’ function; (**B**) the process of model auto-reconstruction; (**C**) pathway mining in a certain model organism; (**D**) gathering experimental and simulated results to identify metabolic engineering targets.

IMGMD (http://imgmd.jiangnan.edu.cn/database/) has a user-friendly website for the following applications: (1) It can be used to download standardised GSMMs; this module integrates model-related information, such as gene–protein–reaction relations, genome information, and references (Fig. 1A). (2) It enables auto-reconstruction of GSMMs; this tool is based on homology alignments, and only sequences that meet a threshold are used for model construction. Additionally, transport proteins and sub-cellular location are identified for further model refinement (Fig. 1B). (3) It can be applied to explore potential pathways; using this function, users can explore the potential pathways from one metabolite to another in a certain GSMM (Fig. 1C). (4) It guides metabolic engineering; the mutation library includes *in silico* and *in vivo* metabolic engineering results, and accordingly, it provides guidance for target searches (Fig. 1D).

All web interfaces of the IMGMD database were tested in various browsers, such as Google Chrome, Mozilla Firefox, Internet Explorer, Opera, and Safari on Windows or Linux platforms. Despite minor differences in appearance, all tools functioned normally in all the tested browsers and on all platforms. Among the browsers tested, Google Chrome and Mozilla Firefox provided the best user experience. Hence, we recommend that users access the database using one of these two browsers.

### The ‘model browse’ function in IMGMD

**Figure 2:**
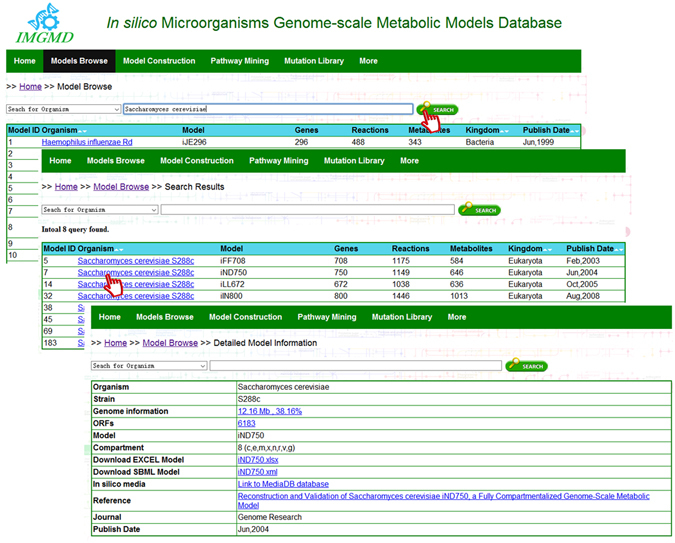
The ‘models browse’ module in IMGMD. Search results for *Saccharomyces cerevisiae* in model browse, and the detailed information on model *i*ND750.

Using model browse, users can browse, search, and download almost all published microorganism models. From the main page of model browse, basic model information, such as the number of genes, reactions, and metabolites can be accessed. Using the search bar, models can be queried by organism name, model name, kingdom, or year of publication. We chose *Saccharomyces cerevisiae* as an example to demonstrate the use of ‘Search for organism’. All 8 *S*. *cerevisiae* models are returned. Then, by clicking on ‘*Saccharomyces cerevisiae* S288c’ for model *i*ND750, a user can find detailed information about the organism (e.g., strain, genome information, and ORFs), model (e.g., model name, cell compartments, model download, and *in silico* media for simulation), and reference (e.g. reference name, journal name, and publication date; Fig. 2). The genome information is linked to the NCBI database^14^, which contains the genome assembly and annotation report for a microorganism. ORFs are linked to the protein sequence downloaded from the UniProt database^15^. The *in silico* media are linked to the MediaDB database^16^, a database of microbial growth conditions in defined media, which can be applied as the constraint condition for metabolic model growth. These standardised GSMMs in IMGMD can be further applied to many analyses using the COBRA Toolbox^17–20^. The ‘model browse’ module attempts to integrate scattered data on organisms, models, and literature, and promotes the establishment of GSMM standardisation.

### The ‘model auto-construction’ function in IMGMD

**Figure 3:**
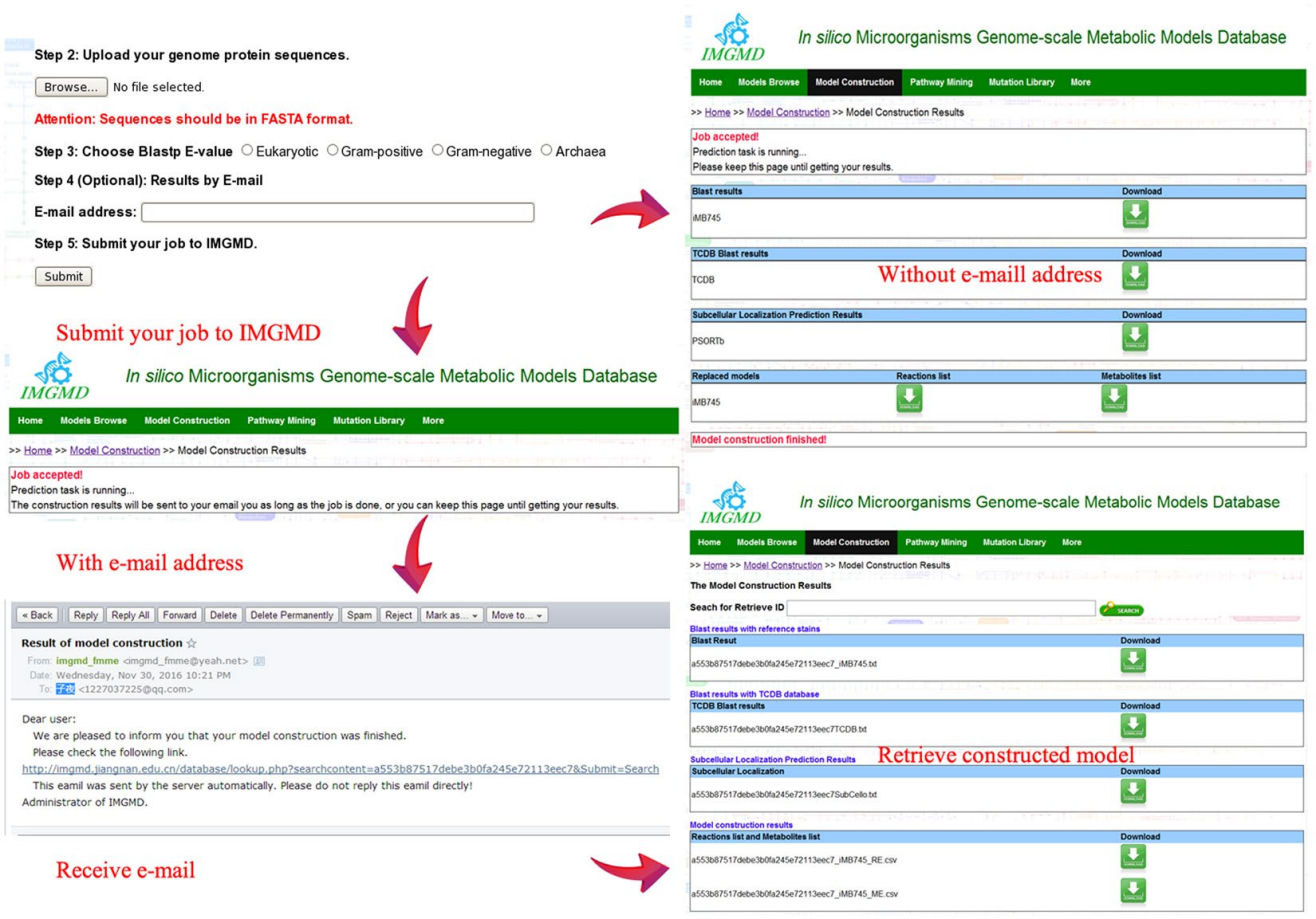
Flow chart for model construction in the IMGMD database.

Five steps are needed to construct a model in IMGMD: (1) choosing three models for reference; (2) uploading the genome sequence; (3) choosing a threshold (eukaryotic: identity ≥40%, identity ≤10E-30; prokaryotic: identity ≥30%, identity ≤10E-6); (4) entering an e-mail address to receive results (optional); (5) submitting the job to the IMGMD database. Once the job is complete, the results contain three parts, including the model, transport proteins, and prediction of protein subcellular localisation (Fig. 3). Model construction is automatically implemented on the basis of the sequence alignment results. After protein sequences are submitted, the local BLASTP program will calculate the sequence similarity. Sequences that meet the established threshold are automatically screened using a Python script written in our lab. Based on the local Blast results, genes with high similarity are replaced in the reference models. Additionally, transport proteins are identified according to the alignment results, using the TCDB database^21^. For eukaryotic organisms, WoLF PSORT^22^ was chosen, whereas for prokaryotic organisms (gram-positive, gram-negative, or Archaea), PSORTb^23^ was employed to predict protein subcellular localisation.

Although some software or platforms for model auto-reconstruction have been developed, including ModelSEED^24^, RAVEN^25^, COBRA Toolbox^26^, SuBliMinal^27^, these tools have their advantages and disadvantages^12^. For instance, ModelSEED (http://modelseed.org/) is a Web service that includes the RAST genome annotation tool. Based on the annotation results, a model for a specific organism can be reconstructed automatically. Given that the RAST service (http://rast.nmpdr.org/rast.cgi) can annotate only prokaryotes, ModelSEED has limited applicability to eukaryotes. Besides, model construction by ModelSEED will take a long time, according to the job numbers. IMGMD is also a web platform that serves for model construction. It is based on the results of genome homologous alignment. Users can upload a target organism’s genome sequence and choose relevant parameters. After submission of the job to IMGMD, results will be returned within 1 day. Nonetheless, a model constructed by IMGMD is a draft model. It still needs to be further processed to obtain a GSMM. The COBRA Toolbox is based on the Matlab platform, which is commonly used for model construction. The COBRA Toolbox requires users to have basic Matlab knowledge and an advanced computer configuration for model analysis (Table 1).

**Table 1:** Selected characteristics of software platforms for reconstruction and simulation of metabolic networks.

	Model SEED	RAVEN	COBRA Toolbox	SuBliMinal	IMGMD
Input	Genome annotated in RAST	Annotated genome sequence	GSMM	Species name	Species genome sequence
Reference Database	SEED	KEGG	N/A	KEGG, MetaCyc	IMGMD
Interface	Web	Matlab	Matlab	Command Line	Web
License	Free	Free (requires a Matlab license)	Free (requires a Matlab license)	Free	Free
Output	SBML, Excel	SBML, Excel	SBML, Excel	SBML	Excel
Supports Simulations	Yes	Yes	Yes	No	No

### Pathway mining function in IMGMD

**Figure 4:**
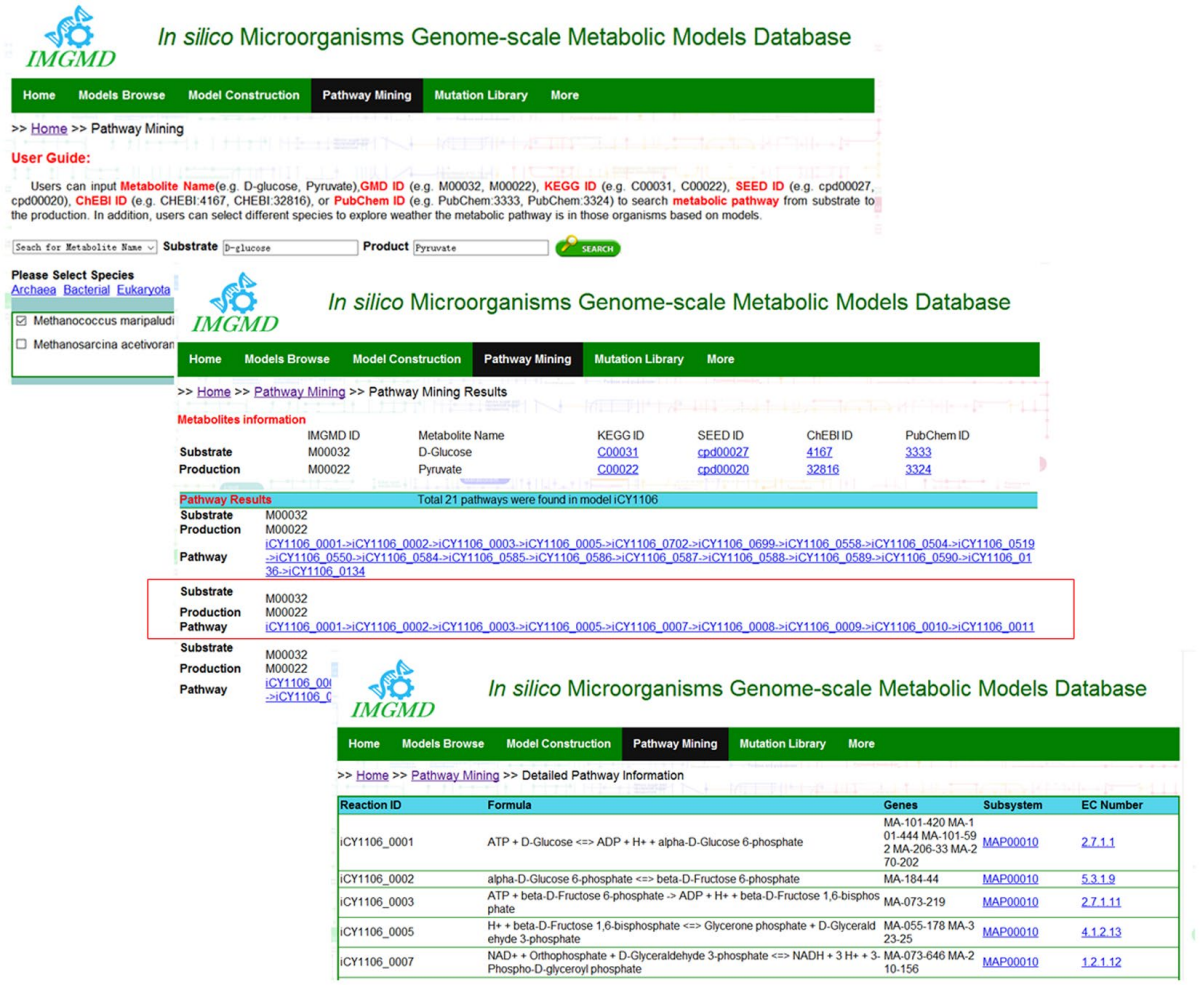
Pathway mining results for the *i*CY1106 model from glucose to pyruvate. A summary of all pathways found in the *i*CY1106 model from glucose to pyruvate, and detailed reaction information for a pathway in model *i*CY1106.

In this module, users can explore metabolic pathways at three levels. (1) According to the input metabolites as substrates and products, total pathways from a substrate to product in a GSMM can be output. For example, in the *Mortierella alpina* model *i*CY1106^28^, 21 pathways exist from glucose to pyruvate, indicating that in addition to the basic glycolysis pathway in *M*. *alpina* (according to the KEGG pathway^29^), other pathways also could generate pyruvate. On the web page of pathway-mining results, information about the substrate and production can be linked to some metabolic databases, like KEGG^29^, ModelSEED^24^, ChEBI^30^, and PubChem^31^. Besides, on the page of detailed pathway information, reactions participating in a pathway are shown, including Reaction ID, Formula, Genes, Subsystem, and EC numbers (Fig. 4). (2) Comparisons between two or more GSMMs help to understand phenotypic characteristics based on metabolic pathway differences. When comparing the pathway differences between two Archaea, *Methanococcus maripaludis* (*i*MM518)^32^ and *Methanosarcina barkeri* (*i*MG746)^33^, there were 8 and 12 pathways from glucose to pyruvate, respectively. (3) Pathways that generate highly valuable products may exist in typical organisms. To mine these potential pathways, users can choose all collected models for the search, and then choose reactions in which species and corresponding genes can serve as references for a target strain to guide strain design. Considering these three levels, the function of pathway mining may be useful in synthetic biology and systems metabolic engineering.

### Mutation library function in IMGMD

The pathway prediction tool enables new pathway design for metabolic engineering; additionally, the mutation library function can be used for optimisation of the host strain. It can help to identify targets that couple cell growth with product formation, e.g., targets for gene upregulation, downregulation, and gene deletion^34, 35^. In IMGMD, a library that combines *in vivo* and *in silico* results to guide metabolic engineering was created.

**Figure 5:**
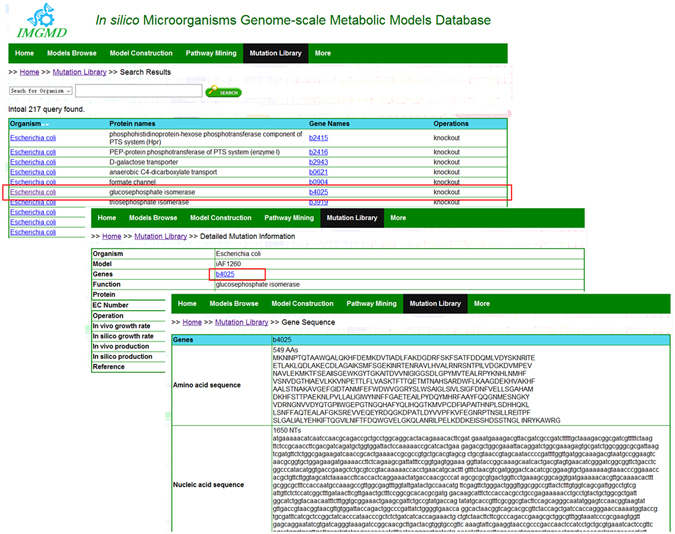
*In silico* and *in vivo* results on the *b4025* deletion in the *i*AF1260 model, and detailed information on *b4025* in *E*. *coli*.

Organisms, models, and genes can be used as keywords to search for mutation information. For example, in a search for mutation information with model *i*AF1260, 217 results can be found. The effect of a knockout of *b4025*, which encodes glucosephosphate isomerase (pgi, EC: 5.3.1.9) in *E*. *coli*, the growth rate and production rate can be viewed on another webpage (Fig. 5). According to the information on this new page, when galactose serves as a carbon source, the *in silico* growth decreases by 36.1%, while the *in vivo* growth rate increases by 12.0%^36^ (Table 2). Furthermore, the amino acid sequence and nucleic acid sequence of gene *b4025* were also included (Fig. 5). The EC number of 5.3.1.9 is linked to BRENDA database for more detailed information.

**Table 2:** Results of the *b4025* deletion in *E*. *coli* collected by IMGMD^36^.

strain	glucose as carbon source				galactose as carbon source			
	growth rate		H_2_ production (mol/mol)		growth rate		H_2_ production (mol/mol)	
	Scaled	OD_600_			Scaled	OD_600_		
	*in silico*	*in vivo*	*in silico*	*in vivo*	*in silico*	*in vivo*	*in silico*	*in vivo*
wide type	1.31	1.31	1.713	1.58	0.83	0.83	1.74	1.48
^Δ^ *pgi*	1.19	0.02	1.740	0	0.53	0.93	1.835	1.16

*pgi* (b4025): encoding glucose-6-phosphate isomerase (EC: 5.3.1.9), which can catalyse d-glucose 6-phosphate into d-fructose 6-phosphate in glycolysis pathway.

In this module, 950 total mutation results were collected by literature mining. Additionally, 885 results (93.2%) were related to various knockout strategies, involving different algorithms, such as OptKnock^19^, GDLS^37^, ReacKnock^38^, DBFBA^39^, BAFBA^40^, and RobustKnock^41^. The remaining results are related to gene upregulation or downregulation^42^. Combined with the pathway mining and mutation library modules, IMGMD can be used to guide systems metabolic engineering, for both pathway screening and for target identification.

## Methods

### Data collection

**Figure 6:**
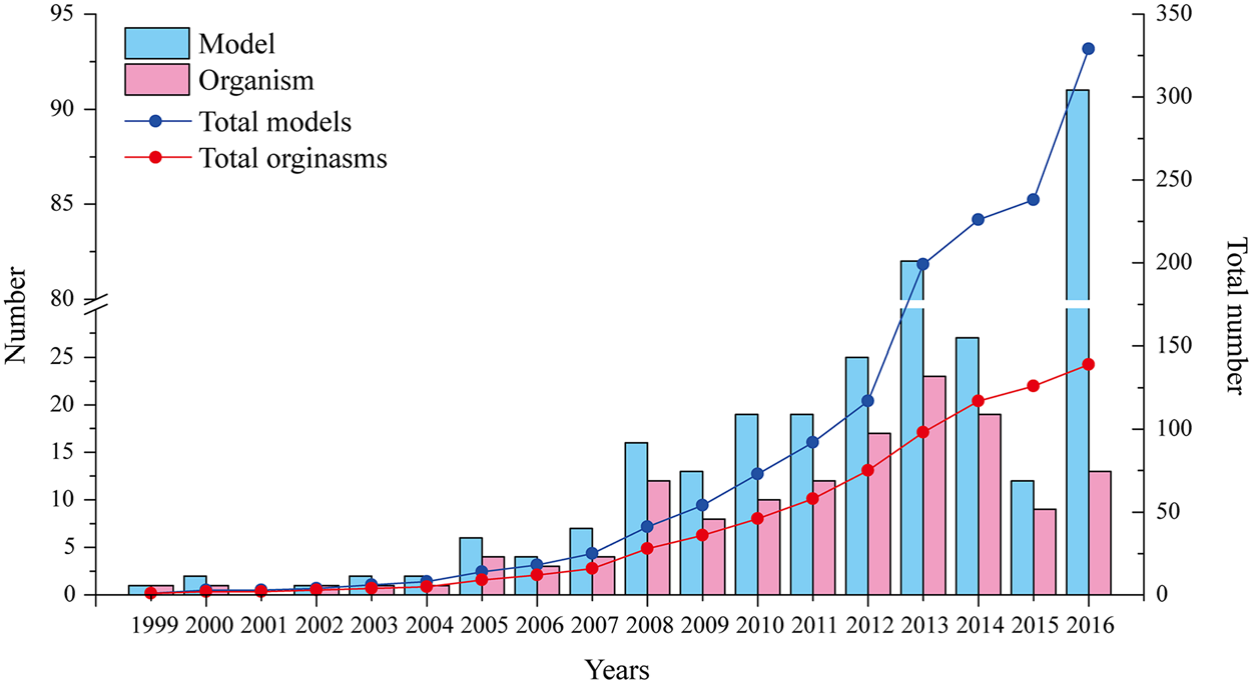
The development of GSMMs from 1999 to 2016.

GSMM data were collected from existing databases (http://systemsbiology.ucsd.edu/InSilicoOrganisms/OtherOrganisms, http://synbio.tju.edu.cn/GSMNDB/gsmndb.htm) and by scrutinizing the primary scientific literature (Web of Science, PubMed, and Google Scholar; Fig. 6). As of November 30th, 2016, 328 GSMMs covering 139 microorganisms have been collected. Of these models, bacteria, eukaryotes, and archaea account for 82.1%, 15.2%, and 2.7%, respectively. Additionally, based on literature mining results from 3,296 articles concerning GSMMs, a mutation library containing 950 mutations in 683 genes from 31 microorganisms was generated.

### Data processing

After collecting information on 328 models, 58 models could not be found, and 270 downloadable models were classified by format according to their written language, i.e., Systems Biology Markup Language (SBML), Microsoft Excel, Microsoft Word, or PDF. To read these models using the COBRA Toolbox, models in all formats were rewritten in the Excel format. Word and PDF files were manually transformed into Excel files. The SBML models were transformed into Excel, using the COBRA Toolbox on the Matlab platform. Nonetheless, during this process, some models written in SBML could not be read using the COBRA Toolbox. Eventually, 265 of total 328 (80.8%) published models were standardised in the Excel and SBML formats and can be downloaded from our database.

GSMMs consist of metabolite lists and reaction lists. Since the GSMMs were constructed by different researchers, metabolites can be represented in various forms. For example, in *E*. *coli* model *i*AF1260^43^, pyruvate was represented as pyr. In *Saccharomyces cerevisiae* model Yeast 1.0^44^, and *Yarrowia lipolytica* model *i*NL895^45^, it was indicated by PYR and s_1277, respectively. In the IMGMD database, according to their unique IDs in various biochemical databases (KEGG, SEED, ChEBI, and PubChem), the metabolites from different models were unified using IMGMD metabolite IDs. Then, 8367 metabolites from these different models were integrated. Additionally, 77.65% of metabolites can be linked to at least one of these databases (Table 3).

**Table 3:** Distribution of metabolites from different metabolite databases.

KEGG	SEED	ChEBI	PubChem	Others*	Numbers	Of total metabolites (%)
√	√	√	√		4133	49.40
√	√	√			4136	49.43
√		√	√		4205	50.26
√	√		√		4296	51.34
	√	√	√		4133	49.40
√	√				4373	52.26
√		√			4212	50.34
√			√		4447	53.15
	√	√			4163	49.75
	√		√		4306	51.46
		√	√		4225	50.50
√					4620	55.22
	√				5649	67.52
		√			4608	55.07
			√		4708	56.27
				√	1871	22.36

^*^Others indicates that metabolites from 235 models could not be found in any of the four databases.

A list of reactions, including the Gene–Protein–Reaction relations for the models, should contain 15 columns of information, e.g., a reaction description, formula, and genes^26^. For the formula column, metabolites are first replaced and rearranged according to their unified IMGMD database metabolite IDs. Additionally, because some information was lacking, data (e.g., gene data) were collected by referring to information such as EC numbers, reaction descriptions, and formulas, in the other columns for the models. Finally, 17 of 19 (89.5%) GSMMs were filled out, except for models of *Streptomyces lividans* (GMD-TK24) and *Pseudomonas putida* (PpuMBEL1071). For the Formula column, metabolites were first replaced with the unified IMGMD metabolite IDs. For example, the reaction catalysed by alcohol dehydrogenase (EC: 1.1.1.1) was unified as ‘M00442[c] + M00003[c] 〈=〉 M00083[c] + M00079[c] + M00004[c]’. In all models, to arrange metabolites on the left and right of ‘〈=〉 ’ in order, a MATLAB script developed in our lab was used. Lastly, the reaction was unified as ‘M00003[c] + M00442[c] 〈=〉 M00004[c] + M00079[c] + M00083[c]’. If we ignore the cell compartments and do not count transport and exchange reactions, the database contains 21436 reactions.

During the process of literature mining, mutation information is stored in an Excel file. Information such as organisms, models, genes, operations, *in vivo* or *in silico* production, and *in vivo* or *in silico* growth rate is collected. Additionally, amino acid and nucleic acid sequences of related genes collected from the KEGG database are also stored in this Excel file.

### Database design and implementation

All processed data are stored in a MySQL database and are available through a Web server built in the standard LAMP (Linux + Apache + MySQL + PHP) system to provide fast and secure data access. XAMPP for Linux 5.6.15 (https://www.apachefriends.org/index.html) was installed on CentOS Linux 5.8 (https://www.centos.org/download/). BLAST 2.2.28 (ftp://ftp.ncbi.nlm.nih.gov/blast/executables/release) and Python 2.7.11 (https://www.python.org/) were used for model auto-reconstruction, and a C++ script based on a depth optimisation algorithm was written to explore the metabolic pathways in a particular GSMM.

## Conclusion

The IMGMD database (http://imgmd.jiangnan.edu.cn/database) provides a platform that integrates the names of metabolites and metabolic reactions from common biochemical databases and existing model repositories. This database includes 328 models for 139 microorganisms and provides 265 standardised models for downloading. Based on a homologous sequence alignment method, models can be reconstructed automatically in the IMGMD database, which can accelerate the process of model construction. Furthermore, IMGMD provides a pathway mining tool for pathway design and a mutation library for strain optimisation.

Compared with other GSMM databases, the IMGMD database is specific for microorganisms. It is user-friendly and feature-rich; accordingly, the scientific community can easily use and extend the knowledge base. Thus, IMGMD will be a useful database for the design phase of systems metabolic engineering. Future developments include integration of the COBRA Toolbox, which will allow users to directly simulate gene deletion or over-expression, on the IMGMD platform. Besides, the IMGMD database is maintained by our lab and will be updated annually, to keep pace with the advances of GSMMs.
